# On the feeling of being different–an interview study with people who define themselves as highly sensitive

**DOI:** 10.1371/journal.pone.0283311

**Published:** 2023-03-17

**Authors:** Marcus Roth, Danièle A. Gubler, Tobias Janelt, Banous Kolioutsis, Stefan J. Troche

**Affiliations:** 1 Department of Psychology, University of Duisburg-Essen, Essen, Germany; 2 Institute of Psychology, University of Bern, Bern, Switzerland; Private University Schloss Seeburg: Privatuniversitat Schloss Seeburg, AUSTRIA

## Abstract

The construct of “sensory processing sensitivity” has become an extremely popular concept outside the scientific literature under the term “high sensitivity” (HS), reflected in a variety of self-help guides and media reports. Therefore, the present study aimed to investigate this phenomenon by examining in-depth individuals who consider the label HS essential to their self-definition. In semi-structured interviews, 38 individuals described their understanding of HS and its perceived manifestations and impact on their lives (among other topics). Subsequently, the data were content-analytically evaluated, i.e., categorized and quantified. One key finding was that HS individuals feel relief following self-attribution or self-diagnosis. Moreover, this self-attribution replaced the feeling of being somehow different from the others, which almost all interviewees mentioned, with positive attributes. The main negative features of HS mentioned were feeling overwhelmed by sensory and emotional stimuli. The results are discussed with regard to the significance of the label HS for this group on the one hand, and with regard to alternative approaches for future research on the other hand.

## Introduction

“Environmental sensitivity” is a generic term for theories proposing first, that people differ in their sensitivity toward both aversive and conducive environments and second, that individual differences exist in the ability to register and process environmental stimuli [1, 2]. Among others, these theories include the theory of “differential susceptibility” which is rooted in developmental and evolutionary psychology and postulates that individual differences in sensitivity represent low versus high plasticity and adaptation [3], the theory of “biological sensitivity to context” which focuses specifically on physiological differences in reactivity [4], and finally, the concept of “sensory processing sensitivity” [5]. The following article deals exclusively with this last aspect.

“Sensory processing sensitivity” (SPS) was introduced by Aron and Aron [5] as a “fundamental individual difference” (p. 347) that affects the way “sensory information is transmitted to or processed in the brain” (p. 347). In addition to the attention SPS has attracted in the scientific literature since that time [see as an overview: 6], the concept has become particularly popular in the non-scientific literature under the term “high sensitivity” (HS), initiated by Aron [7], for whom HS also personally represents an identity-forming characteristic [e.g., 7, 8–11]. Although the present paper is more about everyday understandings of the construct, we will first briefly discuss the scientific conceptualization and operationalization of SPS as well as its inherent problems.

In their theoretical framework, Aron and Aron [5] state that individuals higher in SPS perceive stimuli of lower intensity more easily than others. By contrast, when confronted with stimuli of higher intensity, highly sensitive individuals are more easily overwhelmed and distressed. When Aron and Aron [5] introduced the concept of SPS, one of the main objectives was to distinguish between SPS and the established personality traits of extraversion and neuroticism (emotionality). With regard to extraversion, the authors referred to different personality models, including the arousal theory of extraversion-introversion by Eysenck [12], the behavioral inhibition and activation system (BIS/BAS) by Gray [13], as well as conceptual work on shyness [14]. Aron and Aron [5] assumed SPS to be the underlying basis of *all* these conceptions. Aron and Aron [5] argued that these personality models reflect one common fundamental trait. However, at no point did Aron and Aron [5] state how SPS is specifically related to extraversion. Should SPS serve as a new explanation for extraversion, quasi as an alternative to the arousal theory by Eysenck [12]? Or is SPS conceptualized as a new and unique trait existing alongside extraversion? Unfortunately, Aron and Aron [5] mixed these aspects. Furthermore, the authors did not differentiate between SPS and extraversion in terms of manifestations on a behavioral level. How can one decide what observed behavior is an expression of SPS and what is an expression of extraversion? The rather vague definitions given by Aron and Aron [5] do not allow for such statements [see also vague definitions: 15, 16].

The same problems exist with respect to neuroticism/emotionality. In contrast to extraversion, Aron and Aron’s remarks concerning the relationship between SPS and neuroticism/emotionality are tenuous and restricted to the assumption that high sensory-processing sensitivity causes behavioral styles similar to those considered expressions of emotional instability: “If this sensitivity exists, it would be expected to manifest itself as low sociability and high negative emotionality in some sensitive individuals—the former as a strategy to avoid overstimulation, and the latter as the result of an interaction of the trait with aversive or socially unsupported early experiences involving novel stimuli” [5].

In sum, it turns out to be quite difficult to differentiate between SPS on the one hand and neuroticism/emotionality and extraversion on the other–at least at a behavioral level. As pointed out by Hellwig and Roth [6], it is not even clear whether SPS is a personality trait in the sense of a behavioral style [e.g., 1, 5], or whether it is an ability construct [e.g., 1, 17]. Therefore, it is necessary to sharpen the definition and conceptualization of HSP in the future and to distinguish it more strongly from existing constructs.

Similar difficulties have arisen regarding the operationalization of HSP. Currently, the Highly Sensitive Person Scale (HSPS), a 27-item self-report instrument developed by Aron and Aron [5], is the only available measurement for assessing SPS in adults. Almost all previous studies on SPS are based on this instrument, which has also been adapted into several languages [e.g., 18–22]. While Aron and Aron [5] conceptualize the HSPS as measuring a unidimensional construct, subsequent studies posit the multidimensionality of this instrument. Smolewska, McCabe [23] were the first to state that the HSPS consists of three separate factors, labelled “aesthetic sensitivity” (AES: the awareness of aesthetics; e.g., “Do you have a rich, complex inner life”), “low sensory threshold” (LST: unpleasant sensory arousal; e.g., “Are you bothered by intense stimuli, like loud noises or chaotic scenes”) and “ease of excitation” (EOE: perceived demands; e.g., “Do you get rattled when you have a lot”). Subsequent studies [e.g., 18, 19, 24, 25] also proposed three-factor solutions like the one by Smolewska, McCabe [23].

The HSPS items reflect the conceptual conflation of HSP with established personality constructs—especially neuroticism and introversion, which include classical introversion items (e.g., “Do you find yourself needing to withdraw during busy days into bed or into a darkened room or any place where you can have some privacy and relief from stimulation?”) and neuroticism items (e.g., “Does your nervous system sometimes feel so frazzled that you just have to get off by yourself?”). Besides these traits, the HSPS also includes items capturing other dimensions of the five-factor model [e.g., 26–28], such as consciousness (e.g., “Are you conscientious?”) or openness (“Do you have a rich, complex inner life?”). Thus, at least on the HSPS item level, it appears that SPS is conceptualized as a meta-trait or common basis for nearly all main dimensions of personality. Interestingly, Pluess, Assary [16] argue along this line, whereupon “several of the Big Five personality traits have also been shown to reflect individual differences in Environmental Sensitivity”. However, this might also lead one to assume that the HSPS is nothing but a blended repetition of the Big Five personality traits.

Given the item overlap with scales of the five-factor model, it is not surprising that the HSPS subscales show substantial correlations with Big Five Personality traits. Based on a meta-analysis of studies assessing the associations of the HSPS and its subscales with the Big Five personality traits, Lionetti, Pastore [29] concluded “that SPS is indeed a trait that is relatively distinct from other common personality traits”. However, Lionetti, Pastore [29] did not consider measurement error of the instruments used in their analyses.

To address this issue, disattenuated estimates of the found correlations can be calculated using Spearman’s formula to correct for attenuation. Thus, in the four studies reporting both correlation coefficients between the HSPS and Big Five traits and reliability estimates (Cronbach’s alpha in all cases) for the scales [18, 25, 30, 31], the attenuation-corrected correlations reach r = .87 for EOE and neuroticism and r = -.64 for extraversion and EOE. For LST, the highest correlation is .49 for neuroticism. In contrast, the highest correlation of AES is r = .91 for Openness. These findings suggest a much larger connection between the HSPS and the Big Five traits than previously assumed. Recently, Hellwig and Roth [6] analyzed these relationships at the latent variable level and actually found AES to be identical to the Big Five factor of openness to experience. Furthermore, EOE completely overlapped with the neuroticism facet self-Conscientiousness. LST was the only component of SPS that did not have a correlation with a Big Five Factor close to 1, but still highly overlapped with Neuroticism (*r* = .50) and Introversion (*r* = .38). It seems as if individuals with high SPS scores are characterized by a Big Five profile encompassing high scores in neuroticism, openness, and introversion. Thus, it is necessary for future research on SPS to include the Big Five to avoid replicating all previous findings on neuroticism, extraversion and openness under the label "high sensory processing” (as is lege artis in the jingle-jangle approach). Furthermore, in light of the problematic conceptualization mentioned above, it should be considered whether an alternative SPS instrument should be developed that does not already encompass the Big Five traits at the item level.

Although the SPS concept seems at least questionable from a scientific perspective, the question remains as to why high sensitivity (the popular label) is awarded such enormous importance in everyday life, as reflected, for example, in the large number of popular science books and self-help guides on the topic. These either address the issue in general [e.g., 7, 32] or refer to specific subgroups, such as highly sensitive children [e.g., 9, 33], highly sensitive parents [e.g., 11], highly sensitive boys [34] and men [35], highly sensitive patients [10], highly sensitive employees [36]–and even highly sensitive lovers [8]. Currently, more than 70 guidebooks on the topic of "high sensitivity" have been published in the German language alone. Obviously, there is a market for this topic. Understandably, such guidebooks paint a rather striking picture of this “special species”. Many assertions are also made that find their way back into the scientific literature. These include, for example, the arbitrary adoption of the “orchid assumption” (from the differential susceptibility and biological sensitivity theories, see above) that highly sensitive persons thrive “exceptionally well in ideal conditions” [29], or the assumption that high sensitivity is characterized by “greater depth of information processing” [1, 15]. Until now there is no empirical evidence for either assumption.

Regardless of whether the construct is scientifically meaningful, there seems to be a strong need for individuals to identify themselves with the label "highly sensitive" or adopt it as an identifying characteristic. What attracts these individuals to the label “highly sensitive”? To explain what benefit this self-diagnosis has for these people, it is necessary to ask them. Studies using standardized psychometric questionnaires are not sufficient for this purpose. Furthermore, it is not adequate to select individuals how score high on the HSPS from a larger sample, because for our research question it is not only relevant that the people involved score high on the HSPS but in addition that these people also identify themselves strongly with the label "HS."

Therefore, the aim of the present study is to explore more deeply the mindsets of people who describe themselves as highly sensitive and identify strongly with this label. The focus is on what these people understand under “high sensitivity”, what characteristics they use to make this diagnosis, what effects HS has on their lives, and what the diagnosis actually means to them. To answer these questions, a qualitative approach was chosen involving interviewing the experts on this topic–namely, individuals who use the HS label to identify themselves to a large degree. To our knowledge, this is the first study to systematically explore in-depth people who self-identify as highly sensitive.

## Method

### Data collection and study design

In order to reach individuals who describe themselves as highly sensitive, an advertisement was placed on the website "hochsensibel.org”, the webpage of the “Informations- und Forschungsverbund Hochsensibilität e.V.” (high sensitivity information and research network, registered non-profit association). The advertisement stated that interview partners were sought on the subject of "high sensitivity." It was explained that questions concerning being highly sensitive would be addressed in the context of an interview. Remuneration of 30 Euros was given for participation. Interested individuals were asked to contact the author via e-mail. A total of 83 people responded to the advertisement. Individuals whose responses to the advertisement involved requests for therapy or counselling and individuals who reported being diagnosed with a mental disorder were excluded from further participation. Of the remaining individuals, 40 randomly selected persons were personally contacted by the authors. Individuals were told that they would first participate in a questionnaire study, in which SPS and personality traits were measured (see below), and then be asked to attend an interview appointment. One person cancelled the appointment at short notice, and another person dropped out of the study after the questionnaire. Individuals first completed a survey questionnaire that was administered online. Approximately one to two weeks later, the interview took place.

### Sample

Overall, 38 individuals (male: 13.2%, female: 86.8%) aged between 26 and 68 years (M = 43.7, SD = 10.8) took part in our study. 44.7% of the sample were married, 15.8% were divorced, 21.1% lived in a committed relationship, and 18.4% were single. Participants’ educational backgrounds were distributed as follows: 5.3% had completed compulsory education in Germany (completed the 10th grade), 21.1% had completed an apprenticeship/vocational training, 5.2% had completed a technical college, 18.4% had completed the highest level of secondary education (completed the 12th or 13th grades), and 50% of participants had graduated from a university or university of applied sciences. The study was approved by the Ethics Commission of the Faculty of Human Sciences of the University of Bern (No. 2020-06-00005).

### Measures

#### Questionnaires

To characterize the sample in psychological terms, the following questionnaires were used:

*Sensory processing sensitivity*. Konrad and Herzberg [19] developed a German version of the Highly Sensitive Person Scale, HSPS [5] with three factors: Ease of Excitation (EOE; 10 items, e.g., “I become unpleasantly aroused when a lot is going on around me”), Aesthetic Sensitivity (AES; 5 items, e.g., “I seem to be aware of subtleties in my environment”), and Low Sensory Threshold (LST; 11 items, e.g., “I am bothered by intense stimuli, like loud noises or chaotic scenes”). The items have a 5-point response format ranging from “strongly disagree”(0) to “strongly agree” (4). In the present sample, the internal consistencies were α = 0.86 for the HSPS total score, α = 0.79 for the EOE subscale, α = 0.37 for the AES subscale, and α = 0.86 for the LST subscale.

*Big Five personality traits*. The Big Five personality traits—openness to experience, conscientiousness, extraversion, agreeableness, and emotional stability—were assessed with Ostendorf’s [37] German version of the International Personality Item Pool, IPIP [38], which consists of 50 items, 10 items for each personality dimension. Participants indicate the accuracy of various personal descriptions (e.g., “Am the life of the party”, “Have frequent mood swings”) on a Likert scale ranging from “very inaccurate” (1) to “very accurate” (5). In the present sample, internal consistencies of the scales ranged from Cronbach’s α = 0.70 (agreeableness) to α = 0.88 (emotional stability).

#### Interviews

We conducted 38 semi-structured interviews, ranging from 42 to 128 min in length (M = 84.4 min, SD = 19.3 min). The interviews were held in German. Due to the COVID-19 pandemic, the interviews were conducted via the videoconferencing tool “Zoom”. We developed an interview guide as a foundation for the interviews, which can be found in the S1 File. All interviews started with an introduction containing information about the interview process, i.e., approximate duration, topics, and the use of a guide to structure the interview. Participants were asked to give informed written consent to audio recording for subsequent transcription.

The main body of the interview guide was divided into ten sections, each containing a few open questions regarding the interviewee´s personal view of, experience with, or behavior associated with his/her HS, e.g., “How would you define HS?”. All of these questions were asked in all interviews in the same order and with the same wording, except if the participant used different terminology (e.g., “highly sentient”), in which case the interviewer adapted the questions to match the participant´s own wording. The interview guide also included some optional questions, which further specified each regular question, such as “How do highly sensitive people differ from less sensitive people?”. However, these more specific questions were only explicitly asked if the interviewee had not (implicitly) answered them in their response to the respective main question. After each section, the interviewer briefly summarised the participant´s answers. First, the interviewer asked the interviewee how he/she would define HS and then continued with questions concerning the participant´s self-diagnosis of HS. Most importantly, these included questions about the participants’ individual reasons for describing himself/herself as highly sensitive, the development of the participant´s HS over time, as well as perceived positive and negative aspects of HS. Thereafter, each participant was asked how and to which stimuli he/she reacts particularly sensitively, as well as how he/she deals/dealt with those stimuli. Subsequent topics included questions about the interviewee’s work and its relation to HS, plus questions concerning her/his environment and social contacts, which essentially aimed at the sensitivity of family members, friends, and (if applicable) romantic partner as well as their reactions to the participant´s HS. In addition, the participant was asked if and how HS affects his/her contact with strangers as well as her/his leisure activities.

### Analysis of interview data

First, the audio files were transcribed. Secondly, we anonymized any data that could reveal the identity of the interviewee (e.g., name, place of residence). Then we developed an initial coding guide based on a sample of 10 randomly chosen interviews using the software “QCAmap”. This initial coding guide contained 19 major categories, which were based on the interview questions (see interview guide), such as “How is high sensitivity defined?” or “What is positive about high sensitivity?”. However, due to the large number of questions in the interview guide, not every question could be included in the analysis. So, after forming consensus among the authors regarding the post hoc estimated relevance of each question, some questions were ignored in the analysis, such as “How do you usually feel after a long working day?”. Several subcategories (e.g., “Exhaustion”, “Overwhelming”) were inductively formulated for each major category (e.g., “How does sensitivity manifest itself”) to parsimoniously and exhaustively describe the relevant patterns, similarities, and differences in participants’ answers. Each subcategory was coded dichotomously (0 = not satisfied, 1 = satisfied) for each participant, e.g., the subcategory “Exhaustion” was selected if the participant made statements (at least one) such as “It is just arduous. It is like I have been in an argument. It exhausts you just like that. It saps your energy.” In addition to the 19 major categories, an extra category was created with several inductively defined subcategories, such as “Psychiatric disorder” or “Transmission awareness” (i.e., general importance of talking about high sensitivity). Note that we did not ask explicitly for such information (see interview guide), but inductively created these categories due to observed patterns in the participants’ answers.

Subsequently, we analyzed all 38 interviews based on the initial coding guide. We applied an iterative coding process, i.e., after the creation of a new subcategory, we recoded all interviews with a focus on the added subcategory. If a participant´s statement fell into a subcategory, it was given the corresponding code, regardless of its position in the interview. For example, if one participant stated “After a time, I have the feeling I am getting totally tired, and it all overwhelms me somehow.”, after being asked how he/she would define HS, the subcategory “Exhaustion” was coded as 1, even though the interviewer had not yet asked how the interviewee´s sensitivity manifests itself.

## Results

### Descriptive statistics and comparisons with other studies

Table 1 shows descriptive statistics. To approximate a more or less reasonable characterization of our sample, we selected other studies in which the same instruments were used in German-speaking samples. Preference was given to studies in which the respective instruments were used in unselective–and larger, if possible—samples. The results of these comparisons can also be found in Table 1.

**Table 1:** Descriptive statistics of the present sample and comparison samples.

	Present sample (N = 38)		Comparison Samples		*t* tests			
Variable	*M*	*SD*	*M*	*SD*	*t*	df	*p*	Cohen’s *d*
HSPS-Total	3.12	0.42	1.81^1^	0.69^1^	11.66	1843	< .001	2.29
HSPS-EOE	3.03	0.54	1.86^1^	0.85^1^	9.10	1843	< .001	1.64
HSPS-AES	3.56	0.39	2.38^1^	0.77^1^	10.14	1843	< .001	1.93
HSPS-LST	3.00	0.61	1.51^1^	0.85^1^	10.75	1843	< .001	2.01
IPIP-AGREE	44.74	4.05	39.96^2^	6.12^2^	4.81	8375	< .001	0.92
IPIP-OPEN	42.66	6.14	36.43^2^	5.41^2^	7.08	8375	< .001	1.07
IPIP-EM STA	22.92	7.24	29.56^2^	7.30^2^	5.59	8375	< .001	0.91
IPIP-EXTRA	28.66	7.41	30.52^2^	7.55^2^	1.51	8375	.139	0.25
IPIP-CONSC	38.18	5.59	34.54^2^	6.10^2^	3.67	8375	< .001	0.62

*Note*. HSPS = Highly Sensitive Person Scale; Total: Total score; EOE = Ease of Excitation; AES = Aesthetic Sensitivity; LST = *Low Sensory Threshold; IPIP = International Personality Item Pool; AGREE = Agreeableness*, *OPEN =* Openness to Experience; EM STA = Emotional Stability; EXTRA = Extraversion; CONSC = conscientiousness.

^1^ Online survey [31]: N = 1807 (age: range = 18–97, M = 47.8, SD = 14.4; sex: 56% female; educational levels: no school-leaving certificate = 0.6%, 9 years of school = 11.7%, O-level = 28.1%, A-Level = 23.1%, university degree = 33.2%, doctoral degree = 3.4%).

^2^ Fachkraft 2030 survey [39]: N = 8339 (age: range = 15–37, M = 24.8; sex: 60% females; education level: 9% without university degree, 32% bachelor’ degree; 5% master’s degree or equivalent, 4% other degress).

As might be expected, our HSP sample is characterized by comparatively high scores on the HSPS as well as the subscales of this instrument. 92% of the individuals in our sample fall more than one standard deviation above the mean of the HSPS total score in the study by Schredl, Blamo [31] (EOE: 74%, AES: 92%, LST: 82%). This impressively illustrates that our sample—at least in comparison the unselected sample by Schredl, Blamo [31]–actually contains "highly sensitive persons" as defined in the HSPS by Aron and Aron [5]. With respect to the dimensions of the five-factor model of personality, our sample is more agreeable, more open to experiences, and more conscientious compared to the sample of the “Fachkraft 2030” survey by Seegers, Bergerhoff [39], as well as less emotionally stable. Interestingly, no differences were found with respect to extraversion.

Although the samples from the papers selected allow only approximate comparisons with the sample of the present study, they largely confirm previous findings using unselected samples. First, as expected, the subjects of the present sample score high on the HSPS scale. Second, we found that our sample is characterized by comparatively higher values in openness and neuroticism.

### Qualitative results

Detailed information on the frequencies with which the extracted categories were coded can be found in S1 Appendix.

#### Definition of HS and its manifestation

The coding revealed that 34 participants mentioned an increased or altered response to emotional stimuli as a defining component of HS, e.g., “stimulus satiation, also in the emotional domain. That means, if there is a strained atmosphere, you are not able to really stand that.” Furthermore, 14 of these 34 participants more specifically described an increased or altered empathic response as defining, like “And also when I walk into the room, I feel what the others are feeling.” Additionally, 22 persons gave statements such as “perceiving stimuli more, I guess, also being less able to block them out, which often results in such a stimulus satiation” or “that inner and outer sensory stimuli just slip very crudely and unfiltered and uncategorized into my consciousness”, which characterise HS as lacking the ability to block out stimuli. Expressions like „All stimuli, which, I tell you, stream in on me, I perceive more strongly and process longer, need more time to process and to collate them” are indicative of a further subcategory (More conscious/longer processing of stimuli), which was coded in 18 cases. Another emergent component (13 cases) of the definition of HS was an increased or altered response to sensory stimuli like “loud noises” or “strong solar radiation”. 11 participants mentioned more intensive perception of stimuli (e.g., “Everything just a little keyed up, thus excessive. Everything perceived just a little intensively“), while 9 participants mentioned more detailed perception of stimuli. Note that these subcategories were coded independently of one another, i.e., in most cases, participants made statements that fell into several of these subcategories.

The vast majority of participants (n = 32) mentioned reacting particularly to emotional stimuli, e.g., “No matter whether I read a book or watch a movie or get told something. So, there I feel with the person, yes.”In addition, a majority of participants indicated reacting to noises (n = 32; e.g., “announcements, people jabbering, phone calls”), visual stimuli (n = 28; e.g., “bright light”), odours (n = 25; e.g., “perfume”) and interoceptive stimuli (n = 23; e.g., “hunger” or “tiredness”). 17 participants mentioned reacting to exteroceptive stimuli (e.g., “the cold” or “scratchy cloth”), while only 9 participants reported reacting to (physical) pain (e.g., “headaches”).

Several other characteristics (not stimuli) belonging to HS emerged in the qualitative analysis: The majority of participants (n = 30) made statements in line with the psychological construct of affective empathy (participants may have used different wording, e.g., “compassion” or “noticing more swings compared to others”), while 14 participants made statements related to cognitive empathy (e.g., “putting myself in other people´s positions or being better able to understand others”). Moreover, 23 participants indicated emotionality (e.g., “feeling so extremely deeply”). Less often reported constructs here are closeness to nature (n = 16), perfectionism (n = 14) and creativity (n = 11).

#### Positive and negative consequences of HS

Most participants (n = 24) described intensively perceiving positive stimuli, e.g., walking through the forest or hearing music, as a positive aspect of HS. Further positive facets mentioned were perception of details (n = 12; e.g., “on the street–symbols, signs”) and affective (n = 18; e.g., “I think it is positive that you have understanding”) as well as cognitive empathy (n = 10; e.g., “I can somehow put myself in other people´s positions very well.”).

The most frequently reported negative aspect of HS is exhaustibility or overstimulation (n = 26). For instance, participants described “When you are overexcited, yes. Then it is almost a bit like you are internally imploding” or realizing “that [their] body is really sapped of energy.” Moreover, 16 participants viewed their emotionality as negative about HS; for example, one participant stated: “I have wished I would just have less feelings.” In addition, 16 participants reported negative reactions by others as problematic, including the feeling of being misunderstood or hearing statements like “You are so thin-skinned, you are so complicated.”

Twenty-three participants stated feeling overwhelming, like “feeling easily overwhelmed like that” or “I have the feeling that I will soon be ripped apart and my head will explode”, while 16 persons described exhaustion, e.g., “That is arduous. That just costs a lot of energy.” In addition, 21 participants indicated feelings of tension or panic symptoms, such as a “very strong level of arousal with a racing heart” or “breathlessness”.

Most participants (n = 31) reported evasive strategies of coping with relevant stimuli (e.g., “When I suffer, I withdraw”) as well as relaxation strategies (n = 21; e.g., yoga, autogenic training, or meditation). Only a minority of participants reported behavioural (n = 14; e.g., “I often have headphones and music when I go into such situations, to block out that environment”) or cognitive strategies (n = 11; e.g., “Then I try to imagine that there is some kind of protective cover around me”).

#### First contact with HS and self-diagnosis of HS

Most people learned about HS on the internet (n = 11) or from other people (n = 9) (for comprehensive results for all subcategories, see coding guidelines). The vast majority (n = 32) of participants reported predominantly positive feelings when they first heard about HS, like “That was a true relief” or “Then, however, I thought it is great that I am not alone.” Only one participant described a predominantly negative emotional reaction to first hearing about HS (“That was like–not something else again”). The sudden feeling of being normal (e.g., “Then I realized: Yes, indeed, there is a cause. It is normal that I am like that. Yes, because you always feel a little exotic, like an outsider, if you are different”) was reported 17 times, while 16 participants described that suddenly, their past behaviour made sense (e.g., “I could better understand and contextualize myself”).

21 participants had made a self-diagnosis of HS, e.g., using online tests, while 6 persons received a diagnosis from another person. Furthermore, 25 persons reported that their feeling of being different contributed to the diagnosis.

The most frequently reported change in experience and behaviour due to (self-) diagnosis or knowledge of HS was self-acceptance (n = 30). An example is: “I could accept and embrace myself on another level and completely differently, since before I often also condemned myself for not being so resilient in some things or for working just a little differently, right? “Furthermore, a majority of participants (n = 27) reported paying more attention to their own needs after their diagnosis, e.g., “I try to listen to myself. And if I realize I cannot keep going, then I try to withdraw from the situation, if possible.” Moreover, 21 persons described an increased ability to classify or name misunderstood feelings or behaviours (e.g., “Well, now I can evaluate it. It has surely not decreased–that I react less to these stimuli–but I just can evaluate where it comes from or what it is up to”), while half the participants mentioned having more or better coping strategies now (e.g., “I just try to breathe deeply through the situation. So, trying to stay relaxed in a relaxed way.”). Moreover, 16 participants indicated that their HS has been more or less pronounced depending on life circumstances or life events (e.g. “on vacation”).

#### HS at work and in interpersonal relationships

Most participants indicated working independently (n = 24) as well as a positive working atmosphere (n = 20) as important working conditions. Beyond that, 22 persons stated that HS enriches their work by giving them more empathy, e.g., “that indeed, the patients also feel understood somehow” or “because I can put myself in people´s positions”. In contrast, some participants reported problems at work due to sensory overload (n = 12) or perfectionism (n = 10).

Twenty-five participants were currently in a romantic partnership, of which 16 participants estimated their partners to be less sensitive than themselves. 12 participants reported that their partner had a positive reaction to the participant’s (self-)diagnosis, while only 3 participants reported negative reactions. Furthermore, 15 participants indicated being more sensitive to criticism or rejection in their relationship. Moreover, 22 participants estimated that at least one parent was also highly sensitive. In addition, 25 participants reported problems due to HS in their family of origin, e.g., “I have often not felt seen. Often not supported, had demands placed on me–yes, felt seen–because I had so completely different needs.”, while just 13 described HS enriching their family of origin, e.g., “It was just always practically, because I was always the one who cared about everyone.” Apart from that, 15 participants estimated their circle of friends as similarly sensitive to themselves, while 9 participants rated their circle of friends as less sensitive (n = 1: more sensitive; n = 13: partly more/partly less, i.e., big differences between friends). Furthermore, 22 participants described predominantly positive reactions by their friends to their HS, e.g., “were in large part very understanding”, whereas just one participant mentioned predominantly negative reactions by friends. 24 participants reported that their friendships were enriched by their greater empathy. In addition, 15 participants claimed that HS leads to deeper or closer friendships, while 14 indicated problems approaching strangers due to HS. Most participants tended to spend their leisure time on activities performed somewhat socially (n = 17) or largely socially (n = 6), whereas 8 participants preferred rather social and just one participant largely social activities.

#### Miscellaneous

This superordinate category contains several inductively formulated subcategories that could not be assigned to any major category already reported. To name the most relevant, these subcategories include the feeling of being different (n = 35; e.g., “this feeling of being isolated, even within a group, and being somehow different–I already had this when I was very small”), improved well-being since the insight (that one is highly sensitive; n = 19), as well as the diagnosis of a psychiatric disorder in the past (n = 15). Furthermore, 18 participants formulated the idea that HS is an ability (e.g., “[I] see it as a great gift to have this sensitivity”), and 16 participants (without prompting!) emphasized the importance of talking about HS (e.g., “So, I really like the fact that someone is working on that from a scientific perspective”), which we call “transmission awareness”.

## Discussion

As described in the introduction, the construct known variously as sensory processing sensitivity (SPS) or high sensitivity (HS) has gained enormous importance in everyday psychology, going far beyond the construct’s scientific foundation. As the abundance of popular scientific literature and self-help guides shows, many people identify with and feel that they fall under the category of SPS. Therefore, the present study sought to find out how these people define HS, what manifestations they perceive, and what impact HS has on their lives. For this purpose, we conducted interviews with individuals who strongly define themselves as highly sensitive. Of course, it can be assumed that the definition of HSP and the self-perception of individuals is strongly influenced by social media and popular scientific works. Therefore, it is not surprising that the definitional elements we found are very much reflective of the popular scientific literature [see e.g., 7, 32, 40].

Summarizing the interview statements, the following picture emerges: People see the main characteristic of HS as increased and more intensive perception of emotional and sensory stimuli as well as longer processing of these stimuli. In addition, many subjects describe that they have stronger emotional empathy and are better able to recognize the perspectives of others. This is seen as positive by many, whereas the resulting feeling of exhaustibility and overstimulation is evaluated as negative by most. These data correspond with previous empirical findings that showed that HS is associated with global symptom load [18, 19], stress, [41, 42], and anxiety [43]. Overall, as stated also in the scientific literature cited above, the feeling of being overwhelmed is essential to defining HS [e.g., 5, 17].

Despite these sometimes stressful experiences, almost all of the participants interviewed reported predominantly positive feelings when they first heard about HS. For many, this amounted to an attestation of “being normal”. Many saw themselves as part of a larger community and no longer as outsiders. The feeling of being somehow different from others, which almost all interviewees mentioned, was replaced with positive attributes. Thus, identification with HS can be described as "liberation" from the feeling of being deficient for most participants in this study. Correspondingly, a majority reported greater self-acceptance, especially since many explicitly described HS as a special ability. One participant summarized this connection in a particularly impressive way: “So, if you have always this stamp on your forehead that you are different, then it is very nice to hear that there is a cause for it–that it is not a disorder, but actually a special ability.”

At this point, we would like to make a first attempt to relate this pattern of results to the lack of separability between HS and neuroticism [e.g., 6, 18, 31]: Neuroticism is commonly evaluated negatively, as seen when individuals are asked to report their personality traits under “faking good” instructions [e.g., 44, 45]. This is likely reinforced by terms such as "emotional lability". Here, only the negative side of high neuroticism is considered, while positive features related to increased emotionality are not included—neither in the description of this personality trait nor in the items measuring it. In contrast to traits like “neurotic” or “introverted”, the term “highly sensitive” appears to be positively connotated. Furthermore, this term not only describes deficits, but also includes strengths of high neuroticism. In this way, the concept of HS might be a (quite desirable) way to free neuroticism from its purely deficit-based characterization. As shown by our results, HS people described suffering as a result of the pathologization of their emotionality and therefore experienced the label “highly sensitive” as liberating. In principle, a neutral label for a basic personality trait seems necessary. However, the problem with HS could be that the same mistake, namely judgmental labelling, is now made in the reverse direction: HS is posited as a positive trait by the flower metaphor [1, 29, 46], for example, according to which people are divided into “dandelions” (i.e. low sensitivity), “tulips” (medium sensitivity), and “orchids” (i.e. high sensitivity). Here, it seems useful to find a middle ground in terminology–something between “disturbed neurotics” and “the elected few of the human race”(to put it in rather pointed terms).

Interestingly, a recent study was able to demonstrate links between SPS and both vulnerable and grandiose narcissism [47].

In addition to highlighting people’s need to receive a neutral or positive description of their personality in order to be able to accept themselves, the present study can also advance scientific research. Of course, it remains possible that SPS actually exists as a trait but has so far been insufficiently conceptualized and measured. As mentioned above, it is currently difficult to distinguish HS from neuroticism, introversion and openness. Undoubtedly, one reason for this is the HSPS, which contains a large number of items measuring neuroticism, extraversion, and openness. However, this should not be surprising given how the HSPS items were generated. To extract the basic characteristics of HS people, Aron and Aron [5] asked students from university psychology classes to interview “‘highly sensitive people’—that is, those who are ‘either highly introverted (for example, preferring the company of one or two people) or easily overwhelmed by stimulation (such as noisy places or evocative or shocking entertainment)”. When manifestations of introversion and neuroticism are used as inclusion criteria, it is not surprising that items measuring introversion and neuroticism emerge as a result. It is possible that the “wrong people” were interviewed through this procedure. In contrast, the present study takes a more neutral approach and could serve as a start point for the development of an alternative scale with items that do not measure neuroticism and introversion, but refer primarily to what is specific to HSP.

Nevertheless, the biased sample characteristics can be viewed as limitations of the present study: The vast majority of participants were female and highly educated. These tendencies may not be uncommon in psychological studies, but were especially strong in the current study. However, this is not really surprising due to the recruitment procedure. Furthermore, although N = 38 is considerable for a qualitative sample, this sample size lacks representativeness and therefore must be viewed critically when it comes to generalizability. However, the consistent pattern of our results allows us to assume that the present study’s findings do allow a certain degree of generalization. Of course, such a generalization can only be valid for the German cultural area. Since this is the first study that explores people who define themselves as highly sensitive, information on cultural differences is unfortunately not available. However, the specific ways in which HS manifests “as a blessing and a burden” [47] in different cultures should be an interesting question for future research.

## Supporting information

S1 Checklist Standards for Reporting Qualitative Research (SRQR). (DOCX)

S1 File Interview guide. (DOCX)

S1 Appendix Coding system. (DOCX)
