# Supplementary material for: On the feeling of being different–an interview study with people who define themselves as highly sensitive
Source: PLoS One. 2023 Mar 17;18(3):e0283311. doi: 10.1371/journal.pone.0283311 (PMC10022759; doi:10.1371/journal.pone.0283311)
Supplement: S1 Checklist — (DOCX) [file pone.0283311.s001.docx]

**Standards for Reporting Qualitative Research (SRQR) – Checklist**

| **No.** | **Topic** | **Check** |
| --- | --- | --- |
|  | **Title and Abstract** |  |
| S1 | Title | The title indicates the data collection method (interview) and clearly describes the topic/the focus group (self-identification with *high sensitivity* or people who identify themselves with the construct, respectively). |
| S2 | Abstract | The abstract summarizes the key elements of the study. It includes information concerning its background, purpose, methods, results and conclusions. |
|  | **Introduction** |  |
| S3 | Problem formulation | The phenomenon “Sensory processing sensitivity” (SPS) is introduced, important theoretical and empirical work is cited. The inherent problems of the construct are discussed as well as the concrete issue/focus of the current paper (see S4). |
| S4 | Purpose or research question | At the end of the introduction, it is clarified that the purpose of the present study is to investigate the benefit of the self-identification/the wish to identify oneself with the construct as well as the implicit theories (concerning, e.g., its definition) of people who identify themselves with the construct.  This was realized by interviewing people who strongly identify themselves with the construct. |
|  | **Methods** |  |
| S5 | Qualitative approach and research paradigm | The qualitative approach is the conduction of semi-structured interviews and subsequent coding of participants’ open responses by developing a coding system. Prior to interviewing, an interview guide was developed to make sure each interview is structured and comparable (We have already submitted the interview guide together with the manuscript; see: supporting information – “S1 Supplemental material. Interview guide”). Subsequently, a coding system was developed by searching for commonalities and patterns within participants’ responses (the coding system has also been submitted; see supporting information – “S2 Appendix. Coding system”). A more detailed description of the qualitative approach can be found in the manuscript (pp. 11-12: “Analysis of interview data”). The rationale for choosing the qualitative approach of the present paper – which contradicts the typical approach of studies focusing on the construct of SPS – is the aforementioned (see S4) research question: The purpose is to explore the reason why people (like to) define themselves as *highly sensitive*. This question can hardly be addressed by a quantitative approach, e.g., merely analyzing participants’ responses to the Highly Sensitive Person Scale. Indeed, it requires asking people who are eager to be characterized as highly sensitive (which is achieved by the approach of participant recruitment via an advertisement on the webpage of the (German) *high sensitivity information and research network*). |
| S6 | Researcher characteristics and reflexivity | The present study was not conducted by a single researcher. Instead, several researchers and assistants contributed to each step of the whole process, including interviewing, transcription, coding, etc. Each step was discussed and documented elaborately, the procedure of as well the interview sessions as the coding process was structured to avoid biasing impact of, e.g., one researcher’s personal attributes. None of the contributing researchers or assistants had any personal relationships with any subject. |
| S7 | Context | The interviews were conducted via a videoconferencing tool due to the covid-19 pandemic related policies. |
| S8 | Sampling strategy | An advertisement was placed on the webpage of the (German) *high sensitivity information and research network.* Remuneration of 30 Euros was given for participation. Interested individuals were asked to contact the author via e-mail. Every e-mail was read carefully. Individuals whose responses to the advertisement involved requests for therapy or counselling and individuals who reported being diagnosed with a mental disorder were excluded from further participation. We tried to recruit as many subjects as possible, i.e., we did not exclude potential participants merely due to a stopping rule (“sampling saturation”). |
| S9 | Ethical issues pertaining to human subjects | The study was approved by the local ethics committee (Ethics Commission of the Faculty of Human Sciences of the University of Bern, No. 2020-06-00005). Each participant gave informed written consent before participation. |
| S10 | Data collection methods | The audio track of each interview was recorded (after informed consent of each participant). Subsequently, the audio files were transcribed into written documents and personal data were anonymized. All subsequent analyses were run on these written documents. |
| S11 | Data collection instruments and technologies | The interview guide has been submitted (see: supporting information – “S1 Supplemental material. Interview guide”). The questionnaires are described in the manuscript (see pp. 11-12). The interviews were recorded via a dictation machine. The software *QCAmap* was used for coding (<https://www.qcamap.org>). |
| S12 | Units of study | Demographics of the sample are reported in the method section (see p. 9). Moreover, the sample’s descriptive statistics concerning the Big Five personality traits as well as the Highly Sensitive Person Scale are reported in the results section (see table 1, p. 13). |
| S13 | Data processing | As mentioned, audio files were transcribed into written documents which were then anonymized. |
| S14 | Data analysis | As described in the manuscript (see p. 11-12), 10 randomly chosen interviews were taken for developing the initial coding guide. Subcategories were inductively formulated to effectively describe the relevant similarities and patterns within participants’ responses. An iterative approach was then applied while coding all 38 interviews, i.e., after the creation of a new subcategory, all interviews were recoded with a focus on the added subcategory. |
| S15 | Techniques to enhance trustworthiness | Several researchers collaborated and discussed the development of as well the interview guide as the coding system at every step to avoid individual bias. The raw data as well as the marked passages (i.e., the specifically coded categories) are stored to ensure potential traceability of interpretations. |
|  | **Results/findings** |  |
| S16 | Synthesis and interpretation | The present study provides insight into the implicit theories of people who define themselves with the construct *high sensitivity* (concerning the definition of this construct). Moreover, the current study revealed that most of the people who identify themselves with the construct experienced predominantly positive feelings related to this self-identification. Thereby, this study contributes to the idea that the construct could serve as a positive reappraisal of rather negatively connotated traits (such as neuroticism, see discussion). |
| S17 | Links to empirical data | Quotes are provided in the results section. Moreover, the document containing the coding guide includes detailed results (how many times each category was coded positively). |
|  | **Discussion** |  |
| S18 | Integration with prior work, implications, transferability, and contribution(s) to the field | At the beginning of the discussion, the main findings are summarized. Implications for the field are discussed in detail (see also S16). To our best knowledge, the present study is the first to systematically explore in-depth people who self-identify as highly sensitive. |
| S19 | Limitations | Limitations are discussed at the end of the discussion (e.g., biased sample characteristics). |
|  | **Other** |  |
| S20 | Conflicts of interest | There is no conflict of interest. |
| S21 | Funding | The author received no specific funding for this work. |
