# Supplementary material for: On the feeling of being different–an interview study with people who define themselves as highly sensitive
Source: PLoS One. 2023 Mar 17;18(3):e0283311. doi: 10.1371/journal.pone.0283311 (PMC10022759; doi:10.1371/journal.pone.0283311)
Supplement: S1 File — (DOCX) [file pone.0283311.s002.docx]

**Interview guide (For people who describe themselves as highly sensitive)**

participant code:

**Introduction**:

• A quick check to see how things are going (e.g., meeting place found well)

• Assurance of confidentiality

• Explain the handling of data

• Approximate duration of the interview

• Written consent to audio recording

• Possibly briefly describe the procedure, provide information on the individual thematic

blocks.

• **We work with a guide so that all our interviewers ask the same questions. So, we will**

**structure the interview as well as possible. If you have any comments at the end, we will**

**be happy to take the time.**

• Note that questions may be asked at any time, that breaks may be taken and that answers

are voluntary.

| Note on the interview guide:  **Instruction:**  o The interviewer asks all the questions marked in bold.  o The indented non-bold questions are only asked optionally in case the interviewee did not  answer them.  o After each topic, the interviewer briefly summarises the interviewee's answers.  o The interviewer adapts the terms "since you knew you were highly sensitive" or "since you were diagnosed as highly sensitive" according to the interviewee's terminology. |
| --- |

Thank you for taking part in our study on the topic of high sensitivity and personality. We have invited you because you have indicated in the online study that you are a highly sensitive person. This description is also supported by the answers you gave in the questionnaire for the assessment of high sensitivity. Since there are only a few instruments so far that measure high sensitivity, we want to use this interview to find out in more detail how high sensitivity is described by people who are highly sensitive and how high sensitivity feels in different areas of life.

**Definition of high sensitivity**

First of all, I would be interested to know how you would describe high sensitivity. Then I will ask you some questions about how you found out about high sensitivity and how it affected you.

|  | **Checklist** |
| --- | --- |
| **How would you define high sensitivity?**   - What characterises people who are highly sensitive? Can you describe a specific situation? - How do highly sensitive people differ from less sensitive people? |  |
| **Can you still remember the first time you heard or learned about high sensitivity?**   - Have you read about it/ heard about it on the radio/ seen it on TV? - Did other people talk about it? |  |
| **What was it like for you when you first heard about it?**   - How did you feel about it? - Can you still remember typical thoughts when you first heard about it? - Other people with high sensitivity have reported thoughts such as: "I always knew this." or "At first, I could not do much with it". Do you also know such thoughts? - How did you react when you first heard about it? - Did you further face up to the topic afterwards? If yes, how did you inform yourself? - Have you talked to other people about this? If yes, how did these people   react? |  |

Summary: Did I understand it correctly that...?

**(Self)Diagnosis High Sensitivity**

You have responded to our call and stated that you are a highly sensitive person, or that you have the feeling that you are. I will now ask you some questions about why you describe yourself as highly sensitive. We are particularly interested in your personal assessment.

|  | **Checklist** |
| --- | --- |
| **Why do you think you are a highly sensitive person? What are the reasons for this?**   - Did you come to this conclusion yourself or did another person notice the high sensitivity in you? - If it was another person, can you remember in which situation this   happened?   - Can you tell me a specific situation in which you react particularly sensitively? |  |
| **When did you first notice that you were different in your experience?**   - Can you describe this otherness to me in more detail? - How did this otherness manifest itself? - How did this feel? - Did you always know you were different? - Was there a specific situation that led to this or did this thought come   insidiously?   - Was it like that when you were a child? - Did your family notice something, if so, what? - In what way do other people behave differently from you? |  |
| **Has something changed for you since you knew that this was high sensitivity?**   - Has something changed in your feelings? - Has something changed in your thinking? - Do you behave differently since you found out about it? |  |
| **Has high sensitivity changed for you at times?**   - E.g., was there a time when your high sensitivity was less   pronounced or did not express itself at all?   - And conversely, was there a time when high sensitivity was very pronounced for you? - If so, what do you think was the reason? |  |
| **What do you perceive as positive and what do you perceive as negative about your high sensitivity?**   - What do you perceive as positive about your high sensitivity? - What do you perceive as negative about your high sensitivity? |  |

Summary: You describe that... Did I understand that correctly?

**Values of high sensitivity**

Now I would be interested to know to which situations and to which influences you react particularly sensitively. I will now ask you a few questions about this.

|  | **Checklist** |
| --- | --- |
| **To which stimuli do you react particularly sensitively?**   - Are these external stimuli such as smells, noise or light? - Are these somatic stimuli such as pain, hunger or fatigue? - Are these emotional situations? |  |
| **How does this sensitivity manifest itself for you?**   - How does it feel exactly? E.g., do you feel overstrained or   overwhelmed? Are there also positive aspects?   - Other highly sensitive people also report physical experiences. These can be negative, such as physical tension, or positive, such as energy boosts. Does this apply to you? - What are typical thoughts? - What is typical behaviour? |  |
| **How do you deal with the stimuli to which you react particularly sensitive?**   - What strategies do you use? - Do you tend to withdraw in such moments? - Other highly sensitive people report, e.g., that they do relaxation exercises in stressful situations. Does this also apply to you? - How long does it usually take for this sensation to subside? |  |
| **How has your dealing with such stimuli changed since you knew you were highly sensitive?**   - Which stimuli can you handle better? - Which stimuli are you worse at dealing with? - In which areas of life has your dealing with these stimuli changed in   particular? |  |

Summary: Did I understand it correctly that...?

**Different areas of life**

You have already described that high sensitivity manifests itself in you predominantly through [personalise, see above]. Now I would be interested to know how high sensitivity manifests itself in specific areas of life, such as at work or in a partnership.

**Work**

I will now ask you some questions about your work situation, how you deal with stress and how your high sensitivity manifests itself there.

|  | **Checklist** |
| --- | --- |
| **Can you briefly describe what kind of work you exercise?** |  |
| **To what percentage are you employed there?** |  |
| **Do you like going to work?**   - Which work tasks do you like? - Which work tasks do you like less? |  |
| **How does your high sensitivity affect you at work?**   - Can you describe a specific situation? - Do you have the feeling that, because of your high sensitivity, you manage work tasks less well or better than other work colleagues? - If yes, which ones and why? |  |
| **What do you think would be necessary to work optimally with your high**  **sensitivity?**   - What working conditions? - What strategies? |  |
| **How do you usually feel after a long working day?** |  |

Summary: Do I understand it correctly that...?

**Environment**

Other highly sensitive people report that they have told their family or friends that they are highly sensitive. Now I would be interested to know if you were able to talk to people in your environment about being highly sensitive and how they reacted to it.

|  | **Checklist** |
| --- | --- |
| **Did you talk to other people about the topic of high sensitivity?**   - With which people were you able to exchange information about high   sensitivity?   - What do these people think about it? - How did these people react to this? - Did you find this supportive? - Did you feel confirmed in your assumptions by such conversations? - Did you feel taken seriously? |  |
| **Are there people in your environment who are highly sensitive or have a tendency to be highly sensitive?**   - What relationship do you have with these people? - Do you feel closer to these people than to other people? - Are there similarities between your high sensitivity and the high sensitivity of these people? - If so, how does this similarity manifest itself? - Are there differences between your high sensitivity and the high sensitivity of these people? - If so, how do these differences manifest themselves? |  |
| **Have you been oriented towards close people, actors, musicians or authors who are highly sensitive? (If already mentioned above, pick up here)**   - Can you briefly describe who this person is, what he/she does and why you are oriented towards him/her? - Can you tell me what inspires you about this person or what you like about him/her? |  |

Summary: Did I understand it correctly that...?

**Social contacts - partnership**

Now I would be interested to know how high sensitivity manifests itself in dealing with other people and in your social life.

|  | **Checklist** |
| --- | --- |
| **Are you currently in a partnership?** |  |
| **How sensitive would you rate your partner?**   - Compared to you. |  |
| **Does your partner know that you are highly sensitive? (If already mentioned above, do not repeat)**   - How did your partner react when you told him/her about your high   sensitivity? |  |
| **How does your high sensitivity affect your partnership?**   - Do you think that being highly sensitive affects your relationship? If yes, how? - Do you think that high sensitivity enriches your relationship? If yes, how? |  |
| **How does your high sensitivity affect conflicts in your partnership?**   - Other highly sensitive people report, e.g., that they react very   sensitively to rejection in conflicts in their partnership and thus also feel rejected more quickly. Could this also apply to you?   - Can you handle conflicts in your partnership better since you know that you are highly sensitive? - If so, what could be the reason? What strategies do you use? |  |
| **Has anything changed in your partnership since you knew that you are highly sensitive? If yes, what?**   - Has interaction and communication changed? - Have feelings, such as closeness or affection, changed? - Has the dealing with conflicts in your partnerships changed? - If so, how? |  |

Summary: Did I understand it correctly that...?

**Social contacts - parents & siblings**

Now I ask you some questions about dealing with your parents and siblings.

|  | **Checklist** |
| --- | --- |
| **How sensitive would you rate your parents and your siblings?**   - Compared to you. |  |
| **Do your parents and siblings know that you are highly sensitive? (If already mentioned above, do not pick up again)**   - If so, how did they react when you told them about your high sensitivity? |  |
| **How does your high sensitivity affect your interactions with your parents and siblings?**   - Do you think that your high sensitivity enriches your family? If yes, how? - In which situations does your high sensitivity particularly enrich the family? - Do you think that high sensitivity impairs your family? If yes, how? - In which situations does your high sensitivity particularly impair the family? - How did your parents react to your high sensitivity? - How did this influence your childhood? |  |
| **Has the behaviour of your parents and siblings changed since they knew that you are highly sensitive? If yes, how?**   - E.g., did the interaction or living together change? - Did the relationship with family members change? |  |

Summary: Do I understand it correctly that...?

**Social contacts - friends/acquaintances**

Now I ask you some questions about your contact with your friends/acquaintances/colleagues.

|  | **Checklist** |  |
| --- | --- | --- |
| **How sensitive would you rate your friends?**   - Compared to you. |  |  |
| **Do your friends know that you are highly sensitive? (If already mentioned above, do not pick up again)**   - If so, how did they react when you told them about your high sensitivity? |  |  |
| **How does high sensitivity affect your circle of friends?**   - Do you think that being highly sensitive enriches your friendships? If yes, how? - What do your friends particularly value about you being highly sensitive? - Do you think that being highly sensitive impairs your friendships? If yes, how? - Do you avoid situations like music events because of your high sensitivity? If yes, why and how do you feel about it? - What bothers your friends about you being highly sensitive? |  |  |
| **What is your favourite thing to do with your friends?**   - What does an ideal day with your friends look like? |  |  |
| **Has anything changed in your friendships since you knew you are highly sensitive? If yes, what?**   - E.g., has interaction and communication changed? - Have you lost friendships or made new ones since knowing that you are highly sensitive? |  |  |

Summary: Did I understand it correctly that...?

**Social contacts - strangers**

| **How does high sensitivity affect contact with strangers?**   - Do you think that high sensitivity makes it difficult or easier for you to approach strangers? - If yes, why? - In which situations? |  |
| --- | --- |
| **Has anything changed in your contact with strangers since you knew you are highly sensitive? If yes, what?**   - Has your communication with strangers changed? |  |

Summary: Did I understand it correctly that...?

**Interests and activities**

Now I would like to know more about what interests you, what activities you like to pursue and how high sensitivity manifests itself there.

|  | **Checklist** |
| --- | --- |
| **What interests/hobbies do you have?** |  |
| **What activities are important to you in your free time?** |  |
| **How does high sensitivity affect these interests and activities?**   - Do you think that high sensitivity negatively influences your interests and activities? If yes, how? - Do you think that high sensitivity positively influences your interests and activities? If yes, how? |  |
| **Are there interests that you pursue more or less since you learned that you are highly sensitive?**   - If yes, which ones? - How do you feel since you have been pursuing these interests more or less? |  |

Summary: Do I understand it correctly that...?

**­­**

**Conclusion of the conversation**

- Thank you for the conversation
- Give the opportunity to add something
- Asking how the conversation was experienced
- Ask for suggestions, open questions
- Short information about the further procedure
- Contact opportunities
- Farewell
