# Supplementary material for: On the feeling of being different–an interview study with people who define themselves as highly sensitive
Source: PLoS One. 2023 Mar 17;18(3):e0283311. doi: 10.1371/journal.pone.0283311 (PMC10022759; doi:10.1371/journal.pone.0283311)
Supplement: S1 Appendix — (DOCX) [file pone.0283311.s003.docx]

**Appendix: Coding system**

*Note: Each subcategory was coded dichotomously (0 = not satisfied, 1 = satisfied). The number after each subcategory represents the number of participants whose responses fell into this category.*

**1. How is high sensitivity defined?**

*This is about definitional statements. What does the participant think about what constitutes high sensitivity?*

1.1 No filter, perceiving more stimuli, not being able to block them out 22

1.2 Perceiving stimuli in greater detail (perception of details) 9

1.3 More intensive perception of stimuli 11

1.4 More conscious/longer processing of stimuli (time aspect) 18

1.5 Increased or altered response to sensory stimuli 13

1.6 Increased or altered response to emotional stimuli (emanating from others) 34

(1.6 only: 10, with 1.6a and/or 1.6b: 24)

1.6a Increased/changed empathic resonance to others' emotions 14

1.6b Increased/changed emotional reactions to others' emotions that do not match the other person's emotions 2

1.7 Cognitive empathy 3

1.8 Other 8

**2. How did the person learn about high sensitivity for the first time?**

2.1 Elaine Aron's literature 2

2.2 Newspapers/magazines 1

2.3 Internet, social media 11

2.4 Books 8

2.5 Films/TV shows 1

2.6 Other people 9

2.7 Therapist 4

2.8 Other 1

**3. What was the person’s reaction when he/she first heard about high sensitivity?**

3.1a Predominantly positive feelings (e.g., strong, relieved, interested, excited, etc.) 32

3.1b Predominantly negative feelings (e.g., ashamed, guilty, angry, labelled, etc.) 1

3.1c Neutral feelings (no evaluation) 4

3.2 Revelation (aha moment: suddenly past behaviour makes sense) 16

3.3 Person suddenly feels normal or no longer strange 17

**4. Why does the person think he/she is highly sensitive?**

*Many already name specific stimuli to which they react sensitively or characteristics that fall under high sensitivity here. These are coded in subsequent categories within the coding system. Here is merely coded how the diagnosis arose.*

4.1 Describes that the feeling of otherness contributed to the diagnosis 25

4.2 Self-diagnosis 21

4.2a Tests/questionnaires on the internet 11

4.2b Self-diagnosis: Other form 12

4.3 Diagnosis by other person 6

4.4 Other 3

**5. Changes in experience and behaviour due to self-diagnosis or knowledge of high sensitivity.**

5.1 Now has more strategies (quantitative) or better ones (qualitative) to cope with stimulus overload 19

5.2 Previous behaviour is reinterpreted, makes more sense (embedded in new high sensitivity schema/embedded in own life story). 12

5.3 Since the insight, can better classify or name misunderstood things (stimuli, feelings,
behaviour, etc.) -> increased self-knowledge 21

5.4 Generally pays more attention to own needs/ better ability to set boundaries/ directs focus now to the self -> more self-determination/autonomy 27

5.5 More open communication of one's concerns 8

5.6 Experienced self-acceptance since learning about high sensitivity 30

5.7 Now feels group affiliation 7

5.8 Insight has led to therapy progress/ helped coping with psychological stress 5

5.9 Perceives stimuli in general as subjectively less aversive/disturbing 8

5.10 Directs his/her attention more to stimuli 3

5.11 Other changes 6

**6. Has high sensitivity changed at times?**

6.1 Tried to be less sensitive for longer life phases 7

6.2 Suppresses high sensitivity at work (e.g., tries to be less emotional) 2

6.3 Phases of psychological stress in the past that covary with level of sensitivity 8

6.4 More or less pronounced depending on life circumstances (e.g., stress) or life events 16

6.5 Other 7

**7. What is positive about high sensitivity?**

7.1 Empathy: Cognitive 10

7.2 Empathy: Affective 18

7.3 Intensive perception of positive stimuli 24

7.4 Perception of details 12

7.5 Creativity 5

7.6 Analytical skills: social 0

7.7 Analytical skills: non-social 1

7.8 Self-reflection 0

7.9 Other 13

**8. What is negative about high sensitivity?**

8.1 Exhaustibility/overstimulation/overburdening (in groups or through sensory perceptions) 26

8.2 Neglect of own needs/ difficulty setting boundaries 8

8.3 Emotionality 16

8.4 Negative reactions of others (e.g., to emotionality or otherness) 16

8.5 Perfectionism 7

8.6 Restrictions in everyday life (e.g., in leisure time) 8

8.7 Other 6

**9. To which stimuli does the person particularly react?**

9.1 Noises (background humming, loud music, loud talking) 32

9.2 Visual stimuli (unpleasant light) 28

9.3 Odours (e.g., perfume) 25

9.4 Taste 2

9.5 Interoceptive/ somatic (hunger, thirst, tiredness) 23

9.6 Exteroceptive/ tactile (cold, rough fabric) 17

9.7 Pain 9

9.8 Emotional stimuli 32

9.9 Physical proximity 7

9.10 Other 3

**10. What other characteristics (not stimuli) belong to high sensitivity?**

10.1 Empathy: cognitive (being able to assess people, empathise and take perspective, communicate better, adaptability) 14

10.2 Empathy: affective (intuitively understanding the mood in the room, altruism, not being able to bear others’ suffering, compassion, helpfulness) 30

10.3 Emotionality (vulnerability, fear of rejection, compliancy, conflict aversion, need for harmony, difficulty in emotion regulation, intense emotions, susceptibility to stress) 23

10.4 Attention to detail 7

10.5 Closeness to nature 16

10.6 Creativity 11

10.7 Importance of stability 1

10.8 Perfectionism 14

10.9 Moral values, altruistic behaviour, care behaviour 9

10.10 Increased self-reflection 6

10.11 Other 12

**11. How does sensitivity manifest itself?**

11.1 Exhaustion 16

11.2 Feeling overwhelmed 23

11.3 Headache 11

11.4 Tension and/or panic symptoms (increased heartbeat, shortness of breath, tightness in the chest;) 21

11.5 Stomach pain 4

11.6 Paralysis 0

11.7 Intense vigilance 2

11.8 Energy bursts 6

11.9 Other 11

**12. Dealing with stimuli to which the person reacts particularly sensitively (-> used strategies).**

12.1 Cognitive strategies (detachment from emotion, attention control, positive reappraisal) 11

12.2 Evasion, leaving the situation, escape (leaving the room, getting off the train, walking away, turning away from the stimulus) 31

12.3 Behavioural, active strategies (ending a conversation, asking for lights to be turned off, earplugs) 14

12.4 Relaxation 21

12.5 No strategy/ passive (endure, don't let on) 5

12.6 Other 8

**13. What is important for highly sensitive people at work? (Which work tasks do they like, which ones don’t they like, what would be optimal working conditions?)**

13.1 Freedom, great deal of latitude (no restrictions, rules, bureaucracy) 12

13.2 Flexible work structure 9

13.3 Being able to express creativity 5

13.4 Structure and/or routine 9

13.5 Work alone, work in peace 24

13.6 Less time pressure/ stress 11

13.7 Not taking work home or working for long periods at a time 2

13.8 Flat hierarchies 0

13.9 Positive working atmosphere 20

13.10 Other 16

**14. Work (How does high sensitivity affect the person’s work?)**

14.1 High sensitivity is related to conflict avoidance 1

14.2 Reports problems because of perfectionism/ thoroughness (wants to do everything correctly, take as much time as possible) 10

14.3 Reports that perfectionism/ thoroughness enriches work 5

14.4 Reports problems due to sensory overload (wants to do work in peace, have little interaction, low noise level) 12

14.5 Reports problems with lack of structure by others 4

14.6 Enrichment because participant has more empathy, understanding, consideration 22

14.7 Neglecting one´s own needs or difficulties in setting boundaries in favour of others 6

14.8 Emotionality is seen negatively by others 0

14.9 Creativity enriches work 6

14.10 A lot of personal initiative 1

14.11 Strong need for fairness at work 1

14.12 Other 1

**15. Partnership**

15.1a has no partner 13

15.1b Partner is more sensitive than participant 1

15.1c Partner is similarly sensitive as participant 7

15.1d Partner is less sensitive than participant 16

15.2a Partner’s degree of sensitivity is experienced as enrichment 5

15.2b Partner’s degree of sensitivity is experienced as an impairment 1

15.3a Partner reacts positively to diagnosis of high sensitivity 12

15.3b Partner reacts neutrally to diagnosis of high sensitivity 6

15.3c Partner reacts negatively to diagnosis of high sensitivity 3

15.4a Conflicts in partnership are mainly avoided 6

15.4b Conflicts in partnership are addressed/ discussed preventively/ intensively 4

15.5 Partner’s level of sensitivity affected the break-up of the relationship 0

15.6 More sensitive to criticism or rejection 15

15.7a Emotions burst out in conflict situations 4

15. 7b Emotions are held back in conflict situations 2

15.8 Participant having more empathy, understanding, consideration enriches the relationship 10

15.9 Neglecting one's own needs or difficulties in setting boundaries in favour of the other person 2

15.10 Need for distance/quiet leads to criticism 2

15.11 Open communication in partnership 7

15.12 Emotionality is exhausting/ stressful for partner 5

**16. Family**

16.1a Both parents known, both highly sensitive 6

16.1b Both parents known, one highly sensitive 16

16.1c Both parents known, neither highly sensitive 13

16.1d One parent known, highly sensitive 0

16.1e One parent known, not highly sensitive 1

16.1f Parents unknown 0

16.2 Problems/ difficulties due to high sensitivity in family of origin 25

16.3 Problems/ difficulties due to high sensitivity in current family 4

16.4 Enrichment because of high sensitivity in family of origin 13

16.5 Enrichment because of high sensitivity in current family 7

**17. Friends**

17.1a: Circle of friends is for the most part more sensitive than participant 1

17.1b: Circle of friends is for the most part similarly sensitive as participant 15

17.1c: Circle of friends is for the most part less sensitive than participant 9

17.1d Partly more sensitive/partly less 13

17.2a Talked to several friends about high sensitivity 27

17.2b Talked to one friend about high sensitivity 6

17.2c Has not talked to friends about high sensitivity 3

17.3a Friends react predominantly positively 22

17.3b Friends react predominantly neutrally 3

17.3c Friends react predominantly negatively 1

17.3d Very mixed reactions (positive and negative) 5

17.4 High sensitivity leads to deeper, closer, stronger friendships 15

17.5 Made new friends through high sensitivity 9

17.6 Lost friendships due to high sensitivity 9

17.7 Participant having more empathy, understanding, consideration enriches the friendship 24

17.8 Neglecting one´s own needs or difficulties in setting boundaries in favour of others. 1

17.9 More open communication in friendship 11

17.10 Emotionality is exhausting/ stressful for friends 4

17.11 Sensitivity to sensory stimuli is criticised 5

17.12 Other 13

**18. Strangers**

18.1 Problems approaching strangers, e.g., due to shyness 14

18.2 No interest in approaching strangers 3

18.3 Contact with strangers is exhausting 1

18.4 Difficulties opening up to strangers 1

18.5 Very attentive in contact with strangers/ new people ("switch on recepters", "observe closely"). 11

18. 6 Claims to be able to quickly assess strangers 11

18.7 Enrichment through more empathy, understanding, consideration 5

18.8 High sensitivity leads to open communication during contact with strangers 7

**19. Interests and Activities**

19.1 Largely social 1

19.2 Somewhat more social 8

19.3 Partly social/partly alone 4

19.4 Somewhat more alone 17

19.5 Largely alone 6

**20. General**

20.1 Has the person ever experienced bullying (harassment etc.)? 7

20.2 Did the person report feeling different? 35

20.3 Did the person report feeling special (chosen)? 3

20.4 Did the person report feeling highly intelligent or assume an association (of high sensitivity) with intellectual giftedness? 4

20.5 Has the person had a psychiatric disorder in the past? 15

20.6 Has well-being changed since the insight? 19

20.7 Transmission awareness. General importance of talking about high sensitivity. 16

20.8 High sensitivity is an ability. 18
